# Genes Confer Similar Robustness to Environmental, Stochastic, and Genetic Perturbations in Yeast

**DOI:** 10.1371/journal.pone.0009035

**Published:** 2010-02-03

**Authors:** Ben Lehner

**Affiliations:** 1 European Molecular Biology Laboratory, Barcelona, Spain; 2 Institució Catalana de Recerca i Estudis Avançats, Universitat Pompeu Fabra, Barcelona, Spain; Texas A&M University, United States of America

## Abstract

Gene inactivation often has little or no apparent consequence for the phenotype of an organism. This property—enetic (or mutational) robustness—is pervasive, and has important implications for disease and evolution, but is not well understood. Dating back to at least Waddington, it has been suggested that mutational robustness may be related to the requirement to withstand environmental or stochastic perturbations. Here I show that global quantitative data from yeast are largely consistent with this idea. Considering the effects of mutations in all nonessential genes shows that genes that confer robustness to environmental or stochastic change also buffer the effects of genetic change, and with similar efficacy. This means that selection during evolution for environmental or stochastic robustness (also referred to as canalization) may frequently have the side effect of increasing genetic robustness. A dynamic environment may therefore promote the evolution of phenotypic complexity. It also means that “hub” genes in genetic interaction (synthetic lethal) networks are generally genes that confer environmental resilience and phenotypic stability.

## Introduction

A general property of biological systems is that, despite their complexity, they are often little affected by mutations that inactivate genes. This property, termed mutational (or genetic) robustness, is widespread [1], [2], but only poorly understood [3].

Mutational robustness has important implications for both disease and evolution, as it reduces the phenotypic expression of genetic change. For example, differences in robustness may alter the outcome of a disease mutation. During evolution, increased mutational robustness will decrease the short-term potential for phenotypic change. However, mutational robustness can also increase the long-term potential for evolution, because it facilitates the exploration of genotype space, expanding the ‘phenotypic neighborhood’ available to an organism [4], [5], [6].

Insights into mutational robustness have come from large-scale synthetic lethal screens in model organisms [7], [8], [9], [10], [11]. In these screens, pairs of mutations are systematically combined, and the effects on viability are determined. These screens have shown that for nearly all genes robustness to mutation depends on the continued presence of multiple additional gene products [7], [8], [9], [10], [11], [12]. That is, a strong mutation often has no apparent phenotypic effect only if multiple other genes remain functional. Synthetic lethal screens globally define the abilities of genes to mask (or buffer) the effects of mutations in many other loci [13], [14].

A further important conclusion from synthetic lethal screens has been the realization that certain genes protect an organism from the effects of mutations in many different loci with diverse functional roles [8], [11], [13], [14]. These genes, termed genetic hubs, tend to encode components of chromatin remodeling complexes and molecular chaperones [8], [11], [15], [16], [17].

Despite its implications for ‘evolvability’, it is not clear how mutational robustness itself can evolve, as the conditions under which it can be directly selected are rather limited [18], [19]. In addition to genetic change, organisms are also subject to changes in external conditions (the environment). Further, even in a common environment there is normally extensive stochastic variation among individuals, for example in the concentrations of proteins [20]. Dating back to at least Waddington it has been suggested that mutational robustness may be related to the need to withstand these environmental or stochastic changes [21]. Waddington's idea was based on the intuition that environmental change, stochastic variation, and mutation are likely to have similar effects on an organism, because they act through the same underlying molecular processes [21], [22], [23].

Support for coupling among robustness to different types of perturbations comes from *in silico* experiments using ‘toy-cell’ networks. Here a frequent observation for evolved networks is a correlation in the robustness to different types of perturbation (genetic, stochastic or environmental) [24], [25], [26]. Similarly, simulations suggest a correlated robustness of macromolecules to changes in temperature and to mutation [27], [28]. In addition, the chaperone *Hsp90* is known to confer robustness to both environmental and genetic change [16], and the inhibition of genetic hub genes in *C. elegans* (genes with many genetic interactions) produces highly variable phenotypic outcomes [8]. However, it is not known how generally this coupling among the requirement of genes for genetic, stochastic and environmental robustness applies.

In previous work, Levy and Siegal noted a correlation between the number of genetic interactions known for a *Saccharomyces* gene deletion strain, and the phenotypic variance of the strain [29]. We had also noted this relationship, and in this study I use further global quantitative genetic data to extend this observation, and to show that Waddington's intuition of a coupling among genetic, environmental, and stochastic robustness is well-supported by recent genomic experiments. Across a genome, I find that genes are similarly required for robustness to mutational, stochastic and environmental perturbations.

## Results

### Overview of Datasets

**Figure 1:**
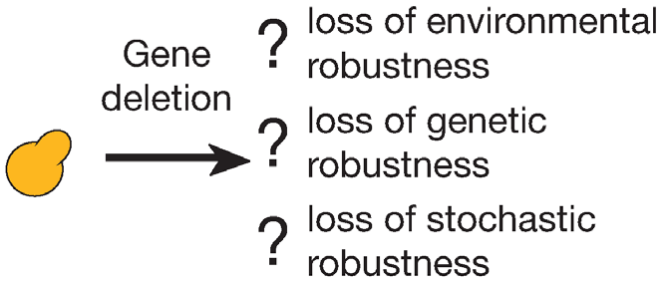
Using global quantitative genetic datasets in yeast to examine the relationships among genetic, environmental and stochastic robustness. The effects of deletions in nearly all non-essential genes on environmental, mutational and stochastic robustness can be estimated using global experiments performed with the haploid gene deletion collection (see materials and methods). This allows one to ask whether gene deletions tend to have similar (i.e. correlated) consequences for the three measures of robustness, or whether they tend to affect genetic, environmental or stochastic robustness independently.

The yeast gene deletion collection provides an excellent resource for globally determining the relationships among environmental, genetic, and stochastic robustness (Figure 1). In each strain a single gene is deleted, and these strains have been tested for growth under diverse environmental conditions. The number of conditions in which a strain shows debilitated growth provides a quantification of its environmental robustness [30], [31]. Further, each strain has been used in synthetic lethal screens, and the proportion of tested mutations which are synthetic lethal or sick in a strain quantifies the strain's genetic robustness [9], [11]. Finally, the overall phenotypic variance of a strain can be quantified from morphological measurements made on large numbers of individual cells [32]. This provides a measure of the stochastic robustness (or phenotypic stability, sometimes referred to as ‘canalization’ [21], [33]) of a strain. These measures, and the data that they derive from, are further explained in the materials and methods section, and allow the effects of mutations on environmental, genetic and stochastic robustness to be compared on a genome-wide scale.

### Gene Deletions Have Correlated Effects on Genetic and Environmental Robustness in Yeast

**Figure 2:**
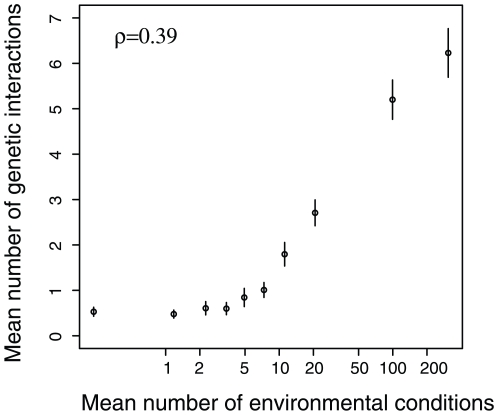
The correlated affects of gene deletions on genetic and environmental robustness in yeast. The effects of mutations on environmental robustness (the number of different environmental conditions in which a gene is required for growth [31]) and mutational robustness (the number of synthetic lethal interactions made by a mutation [9], [11]) are compared across 4656 gene deletions in yeast. Data are plotted for ten equally sized bins of genes. Error bars are +/− one standard error. Spearman rank correlation coefficient (ρ) = 0.39, p<2.2×10^−16^.

First, this data allows the relationship between the effects of gene deletions on genetic and environmental robustness to be examined. Comparing across over 4000 different gene deletions shows that mutations that strongly reduce environmental robustness also generally reduce mutational robustness (Figure 2). That is, if the inactivation of a gene reduces fitness in many different environmental conditions, then it is also very likely to be synthetic lethal with mutations in many different loci. Further, the effects of gene deletions on environmental and genetic robustness are strikingly correlated across all genes (Spearman rank correlation coefficient ( ρ) = 0.39, p<2.2×10^−16^, n = 4656 genes).

### The Coupling Between Genetic and Environmental Robustness Is a General Result

**Figure 3:**
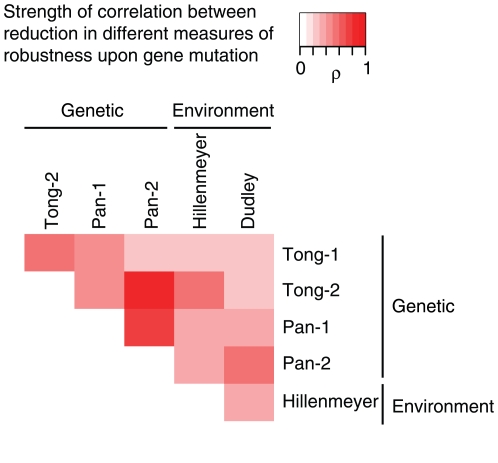
The correlation between the requirement of genes for mutational and environmental robustness is confirmed using multiple different datasets. Correlation coefficients between the effects of mutations on measures of robustness are represented as a heat-map. Datasets: genetic robustness–proportion of synthetic lethal interactions from Tong [11], Pan [9]; environmental robustness–number of environmental conditions in which a strain is required for growth from Hillenmeyer [31], and Dudley [30]. ‘1’–synthetic lethal degree for ‘target’ genes, ‘2’–synthetic lethal degree for ‘bait’ genes. See Table 1 for P-values.

The correlation between the effects of mutations on genetic and environmental robustness is a general result that is observed using independent datasets derived from multiple laboratories (Table 1). In Figure 3 the strength of correlation among the effects of mutations on multiple measures of genetic and environmental robustness are presented as a heat map. Comparing the strength of correlation between different measures of genetic or environmental robustness shows that these are of similar strength to the correlations between different measures of the same type of robustness (i.e. comparing between different measures of either genetic or environmental robustness, Figure 3, Table 1). This shows that much of the discrepancy among measures is likely due to experimental noise (false positive and false negative data points). Further, strong coupling is still observed if all environmental conditions involving small molecule inhibitors are removed from the environmental robustness dataset (ρ = 0.34, p<2.2×10^−16^, small molecules may act analogously to gene deletions).

**Table 1:** Genes confer correlated robustness to genetic, environmental and stochastic perturbations in yeast.

		Genetic			Environmental		Stochastic
		Tong-baits	Pan-targets	Pan-baits	Hillenmeyer	Dudley	Levy/Ohya
Genetic	Tong-targets	ρ = 0.56	0.41	0.25	0.28	0.27	0.18
		P = 1.32×10^−10^	<2.2×10^−16^	0.040	<2.2×10^−16^	<2.2×10^−16^	<2.2×10^−16^
		n = 117	4718	71	4656	4718	4680
	Tong-baits		0.46	0.85	0.51	0.29	0.48
			4.62×10^−7^	9.85×10^−6^	1.77×10^−8^	0.0021	1.16×10^−7^
			117	20	114	117	117
	Pan-targets			0.75	0.37	0.35	0.21
				2.20×10^−13^	<2.2×10^−16^	<2.2×10^−16^	<2.2×10^−16^
				71	4656	4718	4680
	Pan-baits				0.34	0.52	0.30
					0.0056	7.61×10^−6^	0.014
					71	71	71
Environmental	Hillenmeyer					0.39	0.22
						<2.2×10^−16^	<2.2×10^−16^
						4656	4653
	Dudley						0.18
							<2.2×10^−16^
							4680

Spearman Rank Correlation coefficients (ρ), P-values (P) and number of genes considered (n) comparing different measures of genetic, environmental, and stochastic robustness for gene deletions in yeast. Datasets are referred to by first author–Tong [11], Pan [9], Hillenmeyer [31], Dudley [30], Levy/Ohya [29], [32].

Taken together, these data indicate that in general genes are similarly important for buffering environmental and genetic change in yeast.

### Genetic and Environmental Robustness Are Both Also Coupled to Stochastic Robustness

The stochastic robustness of a yeast strain is more difficult to quantify. However, the data from Ohya et al. (2005) provide one measure of this–quantifying the inter-individual variance in a large number of cell morphology parameters, also referred to a the phenotypic stability or canalization [32]. Here I adopt the metric of Levy and Siegal which corrects for mean-dependencies in the coefficient of variation measures for each morphological phenotype, and uses the residual variance in the top 35 out of 70 independent phenotypes to provide an overall quantification of the phenotypic stability of a deletion strain [29]. However, using different measures of inter-individual morphological variation does not change the conclusions presented here (data not shown).

**Figure 4:**
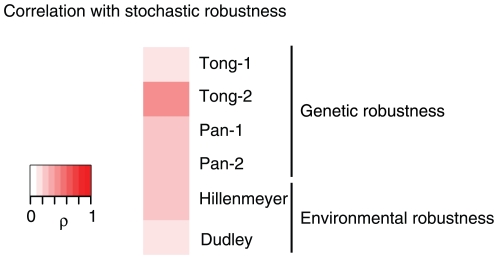
The effects of mutations on mutational and environmental robustness are also correlated with reductions in stochastic robustness. Stochastic robustness is quantified as the variability of cellular morphology among individuals, as described in materials and methods [29], [32]. The correlation of each measure of environmental or genetic robustness across mutant strains with morphological variability is shown. See Table 1 for P-values. Datasets used are as described in Figure 3.

As noted by Levy and Siegal, comparing across all gene deletions, there is a striking correlation between the phenotypic variance of a strain and the number of synthetic lethal interactions known for that strain (Figure 4). Further, phenotypic variance is also related to the robustness of a strain to environmental change (Figure 4). These relationships are confirmed using multiple different genetic and environmental robustness datasets (Table 1).

Taken together, we can say that deletions in non-essential genes in yeast tend to have correlated effects on mutational, environmental, and stochastic robustness. This means that genes that protect the organism from environmental or stochastic change also buffer the harmful effects of mutations, and to a similar extent.

## Discussion

### A Global Coupling Among Genetic, Environmental, and Stochastic Robustness

Mutational robustness is a general property of biological systems, but how and why it evolves is unclear [18], [19], [22], [34]. Here I have shown that inactivating mutations in yeast generally have correlated effects on mutational, environmental, and stochastic robustness. This result, which uses quantitative data from deletions in nearly all non-essential genes, is in striking agreement with the ideas of Waddington and others from the pre-molecular era of biology [21], [22]. Waddington envisaged that organisms would be similarly resistant to environmental, stochastic, and genetic change, because each type of perturbation would act through the same (at that time unknown) underlying molecular processes [21], [33]. The global quantitative data for yeast shows that, as a general principle, Waddington's idea was correct.

### The Evolution of Mutational Robustness

**Figure 5:**
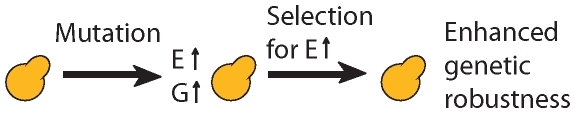
A model for the evolution of mutational robustness. The coupling between the requirement of genes for genetic (‘G’) and environmental (‘E’) robustness means that during evolution, selection for adaptive increases in environmental resilience may often have the side-effect of increasing mutational robustness.

The coupling among genetic, environmental and stochastic robustness means that during evolution selection for adaptive increases in one type of robustness may frequently also increase robustness to the other two types of perturbation. Theoretical work suggests that the conditions in which mutational robustness can be a direct target of natural selection are rather limited [18], [19]. In contrast, selection for increased environmental resilience is straightforward as this can have immediate fitness benefits. During evolution, therefore, mutational robustness may generally evolve as a byproduct of selection for environmental resilience, or possibly for stochastic phenotypic stability [18], [19], [22], [34]. This effect is seen in *in silico* evolution experiments [24], [25], [26], and suggests that evolution in a dynamic environment may promote the emergence of phenotypic complexity (Figure 5).

### Genetic Hubs Are Genes That Buffer Environmental and Stochastic Change

Finally, the coupling among genetic, environmental and stochastic robustness also helps to rationalize one important result from synthetic lethal screens. In these screens, a subset of genes–termed ‘genetic hubs’–are found to buffer the effects of mutations in many functionally diverse loci [8], [11]. Given the results presented here, we can say that these ‘hub’ genes in genetic interaction networks tend to be genes that buffer the effects of environmental and stochastic change. Similar to *hsp90*, therefore, their behavior as genetic hubs is most likely to be a byproduct of a primary evolved function to confer environmental and stochastic robustness.

Most importantly, the results presented here show that, in general, genes that confer robustness to environmental or stochastic perturbations also confer robustness to genetic change, and with similar efficacy. This has important implications for understanding how mutations combine to cause disease, and how the potential for evolution (evolvability) itself evolves.

## Materials and Methods

### Mutational Robustness

Genes required to buffer the phenotypic consequences of mutations in other loci were identified using synthetic lethal genetic interaction screens. A synthetic lethal interaction occurs when the combination of two mutations has a synergistic effect on viability. In these screens a strain carrying a loss-of-function mutation in a single gene is crossed into a panel of strains each carrying a deletion in a single non-essential gene. Double mutant combinations showing synthetic growth or viability defects then identify loci that are buffered by expression of the test gene. Two datasets are used that are derived from different experimental protocols (competitive and parallel growth) performed in different laboratories [9], [11]. In each dataset ‘bait’ and ‘target’ genes are considered separately, because in each case a different number of possible interactions is tested.

### Environmental Robustness

Genes required for growth in different environmental conditions were identified in two systematic genetic screens [30], [31] using the complete set of viable haploid gene deletion strains [1]. For the Hillenmeyer dataset a z-score P-value cut-off of 10^−5^ is used to identify growth defective strains, as recommended [31].

### Stochastic Robustness

The robustness of the same haploid gene deletion strains to stochastic perturbation is quantified using the variance of morphological phenotypes in a single environment [32] quantified with the metric of Levy and Siegal. Here mean-dependencies of coefficient of variation measures are accounted for, and the residual variance in the top 35 out of 70 independent phenotypes provides a measure of the overall decrease in phenotypic stability of a strain [29].
