# Correction: Genes Confer Similar Robustness to Environmental, Stochastic, and Genetic Perturbations in Yeast

**DOI:** 10.1371/annotation/1f52efd2-4d54-428c-9e03-42f1b2b03af0

**Published:** 2010-02-10

**Authors:** Ben Lehner

The affiliations listed for the author are incorrect. The correct affiliations should read: 1 European Molecular Biology Laboratory (EMBL) - Centre for Genomic Regulation (CRG) Systems Biology Unit, Barcelona, Spain, 2 Institució Catalana de Recerca i Estudis Avançats (ICREA), Centre for Genomic Regulation (CRG), Universitat Pompeu Fabra (UPF), Barcelona, Spain. 

